# Supplementary material for: The Cat–Owner Relationship: Validation of the Italian C/DORS for Cat Owners and Correlation with the LAPS
Source: Animals (Basel). 2022 Dec 24;13(1):69. doi: 10.3390/ani13010069 (PMC9817682; doi:10.3390/ani13010069)
Supplement: Supplementary file 1 [file animals-13-00069-s001.zip › animals-2089692-supplementary.pdf]

Table S1. Demographic information of the cats

Demographic Factor	Mdn (Min-Max)
**Age (years)**	8 (0.5-25)
**Age at adoption (years)**	0.25 (0.25-25)
	**% (N)**
**Sex**	
Female	52.2(618)
Male	47.8(565)
**Neutering Status**	
Neutered	96.4 (1140)
Intact	3.6 (43)
**Age when neutered**	
Before 3 years	91.6(1062)
After 3 years	4.7(54)
Never	3.7 (43)
**Breeds**	
Mixbreed	90.5 (1070)
Ragdoll	1.5 (18)
British	0.8 (10)
Maine Coon	0.8 (9)
Norwegian Forest Cat	0.8 (9)
Persian Cat	0.8 (9)
Siamese	0.8 (9)
Siberian	0.8 (9)
Thai cat	0.5 (6)
Chartreux	0.4 (5)
Scottish fold	0.3 (4)
Other	1.9 (23)
**Origin of the cat**	
Professional Breeder	2.7 (32)
Private (e.g., Friends, acquaintances, family breeder)	29.3 (347)
Given as a gift	3.6 (42)
Born at home	4.0 (47)
Pet shop	0.1 (1)
Found/Shelter/Sanctuary	60 (710)
**Walking with the cat**	
With a leash	3.9 (46)
Without a leash	13.0 (153)
I don’t walk with my cat	82.5 (973)
Other methods of containment	0.6 (7)
**Health issues**	
Yes	21.6 (255)
No	78.4 (928)
**Behaviour issues**	
Yes	9.9 (117)
No	90.1 (1066)
**Living Spaces**	
House and garden	40.1 (474)
House and Terraces/Balconies/Roofs	30.3 (355)
Exclusively in the house	29.1 (345)
Predominantly in the house but with access outdoor	0.17 (2)
Predominantly in the garden but with access indoor	0.17 (2)

**Other Pets**	
None	30.3 (358)
Only dogs	10.7 (127)
Only cats	39.0 (461)
Dogs and cats	20 (237)

Table S2: Results of C/DORS EFA and factor loading including items below cut off. Removed items are in bold.

Items	Loadings		
	**Pet- Owner Interactions (POI)**	**Perceived Emotional Closeness (PEC)**	**Perceived Costs (PC)**
26 How often do you pet your cat?	0.82	-0.08	-0.04
21 How often do you cuddle your cat?	0.82	-0.04	0.01
15 How often do you talk to your cat?	0.66	0.01	0.06
9 Ho often do you spend time enjoying watching your cat?	0.64	-0.02	0.05
30 How often do you hug your cat?	0.61	0.20	-0.07
4 How often do you kiss your cat?	0.48	0.24	-0.02
23 How often do you have your cat with you while relaxing, e.g., watching TV?	0.43	0.22	0.02
7 How often do you play games with your cat?	0.41	0.08	0.04
24 My cat is there whenever I need to be comforted.	-0.05	0.86	-0.02
18 If everyone else left me, my cat would still be there for me.	-0.06	0.79	-0.04
20 My cat helps me get through tough times.	0.04	0.71	0.01
22 My cat provides me with constant companionship.	0.08	0.66	0.03
32 My cat is constantly attentive to me.	-0.03	0.65	0.08
17 I would like to have my cat near me all the time.	0.16	0.54	0.068
5 I wish my cat and I never had to be apart.	0.16	0.48	0.08
25 How traumatic do you think it will be for you when your cat dies?	0.16	0.42	0.01
2 My cat gives me a reason to get up in the morning	0.08	0.39	0.08
13 How often do you tell your cat things you don't tell anyone else?	0.27	0.31	-0.01
10 It is annoying that sometimes I have to change my plans because of my cat.	0.004	-0.01	0.75
8 It bothers me that my cat stops me doing things I enjoyed before I owned it.	-0.05	0.03	0.69
6 My cat makes too much mess.	0.01	-0.04	0.56
11 My cat costs too much money.	0.03	-0.04	0.50
3 There are major aspects of owning a cat I don't like.	0.000	0.14	0.44
1 How hard is it to look after your cat?	0.03	0.03	0.42
**12 How often do you buy your cat presents?**	**0.24**	**0.14**	**0.05**

**28 How often do you give your cat food treats?**	**0.24**	**-0.02**	**0.04**
**31 How often do you groom your cat?**	**0.32**	**0.07**	**0.04**
	**POI**	**PEC**	**PC**
**Variance explained**	14%	14%	7%

Table S3: Descriptive statistics of removed items

Item	Mean	Standard Deviation	Median	Skewness	Kurtosis
**CDORS 12**	2.17	0,95	2	0.74	0.51
**CDORS 14**	1.30	0.81	1	3.23	11.36
**CDORS 16**	1.19	0.72	1	4.50	21.73
**CDORS 19**	1.04	0.34	1	12.0	173.03
**CDORS 27**	1.22	0.71	1	4.67	25.09
**CDORS 28**	3.08	1.44	3	-0.16	-1.08
**CDORS 29**	1.50	0.71	1	2.27	9.96
**CDORS 31**	3.87	1.58	4	-0.20	-0.76
